# Sleep Architecture When Sleeping at an Unusual Circadian Time and Associations with Insulin Sensitivity

**DOI:** 10.1371/journal.pone.0072877

**Published:** 2013-08-08

**Authors:** Hanne K. J. Gonnissen, Claire Mazuy, Femke Rutters, Eveline A. P. Martens, Tanja C. Adam, Margriet S. Westerterp-Plantenga

**Affiliations:** Department of Human Biology, Nutrition and Toxicology Research Institute Maastricht, Maastricht University, Maastricht, The Netherlands; Paris Institute of Technology for Life, Food and Environmental Sciences, France

## Abstract

Circadian misalignment affects total sleep time, but it may also affect sleep architecture. The objectives of this study were to examine intra-individual effects of circadian misalignment on sleep architecture and inter-individual relationships between sleep stages, cortisol levels and insulin sensitivity. Thirteen subjects (7 men, 6 women, age: 24.3±2.5 y; BMI: 23.6±1.7 kg/m^2^) stayed in a time blinded respiration chamber during three light-entrained circadian cycles (3x21h and 3x27h) resulting in a phase advance and a phase delay. Sleep was polysomnographically recorded. Blood and salivary samples were collected to determine glucose, insulin and cortisol concentrations. Intra-individually, a phase advance decreased rapid eye movement (REM) sleep and slow-wave sleep (SWS), increased time awake, decreased sleep and REM sleep latency compared to the 24h cycle. A phase delay increased REM sleep, decreased stage 2 sleep, increased time awake, decreased sleep and REM sleep latency compared to the 24h cycle. Moreover, circadian misalignment changed REM sleep distribution with a relatively shorter REM sleep during the second part of the night. Inter-individually, REM sleep was inversely associated with cortisol levels and HOMA-IR index. Circadian misalignment, both a phase advance and a phase delay, significantly changed sleep architecture and resulted in a shift in rem sleep. Inter-individually, shorter REM sleep during the second part of the night was associated with dysregulation of the HPA-axis and reduced insulin sensitivity.

**Trial Registration:** International Clinical Trials Registry Platform NTR2926 http://apps.who.int/trialsearch/

## Introduction

Homeostatic and circadian processes control the quality of wakefulness and sleep. A primary role of the circadian clock is to promote wakefulness during the internal biological day, and to facilitate the consolidation of sleep during the internal biological night [1–3]. Consequently, misalignment between internal circadian time and wakefulness-sleep schedules leads to impaired wakefulness and sleep disturbance. Circadian misalignment may occur during shift work, jet lag or certain circadian rhythm disorders [4,5]. One of the effects of circadian misalignment is a reduction in total sleep time, but circadian misalignment may also affect sleep architecture. Human sleep is not a homogeneous state, but is composed of rapid eye movement (REM) sleep and non-REM sleep. The non-REM sleep can be further divided into 4 stages of progressively deeper sleep. Stages 3 and 4 of non-REM sleep are also named slow-wave sleep (SWS). The preferential distribution of REM sleep toward the latter part of the night is thought to be linked to a circadian oscillator. Contrarily, the preferential distribution of SWS in the beginning of a sleep episode is thought to be mediated by homeostatic processes, i.e. the length of prior wakefulness [6]. Thus, the circadian phase at which sleep occurs may affect the distribution of sleep stages. Therefore, the first aim of our study was to examine the different intra-individual effects of a phase advance and a phase delay on sleep architecture.

Previous studies have suggested that circadian misalignment may lead to adverse metabolic and cardiovascular consequences, which in turn may result in obesity, diabetes and cardiovascular disease [7–9]. In previous work, we showed that circadian misalignment, both a phase advance and a phase delay, resulted in a concomitant disturbance of the glucose-insulin metabolism and substrate oxidation [10], supporting this hypothesis. Additionally, we have shown in a study on sleep and metabolic consequences that inter-individual changes in sleep architecture, rather than total sleep time, are related to endocine and metabolic parameters [11]. The present study aims to establish if the metabolic consequences of circadian misalignment are connected with the effects of circadian misalignment on sleep architecture. Therefore, our second aim is to examine the inter-individual relationships between different sleep stages, cortisol levels as indicator of HPA-axis activity and HOMA-IR index as indicator of insulin sensitivity.

## Methods

### Ethics Statement

All procedures were carried out with adequate understanding and subjects provided written informed consent at the start of the first test day. The study was conducted according to guidelines laid down in the Declaration of Helsinki and the Medical Ethical Committee of Maastricht University Medical Center approved all procedures involving human subjects. The study was registered, ICTRP registration number is NTR2926. The protocol described here in this study deviates from the trial protocol approved by the Medical Ethical Committee of Maastricht University as it comprises only a part of the approved trial protocol. The protocol for this trial and supporting CONSORT checklist are available as supporting information; see Checklist S1 and Protocol S1.

### Subjects

**Figure 1:**
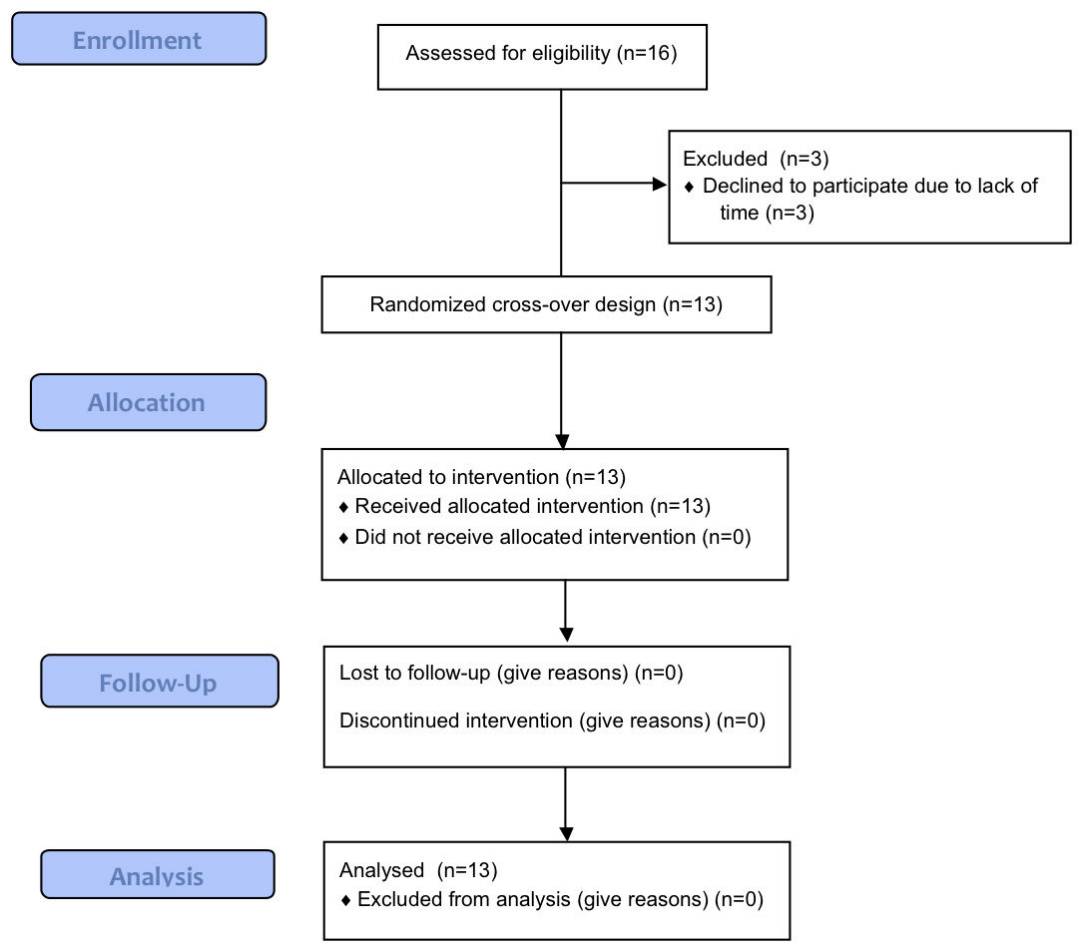
Flow diagram (CONSORT).

Thirteen healthy subjects (seven men, six women) with a mean age of 24.3 years (SD, 2.5) and with a mean BMI of 23.6 kg/m^2^ (SD, 1.7) participated in the present study (Figure **1**). They were recruited via advertisements on notice boards at the Maastricht University. The subjects underwent an initial screening including measurements of body weight and height and completed a questionnaire related to health, use of medication, smoking behavior, alcohol consumption, physical activity, eating behavior and food allergies. All subjects were in good health, non-smokers, not using medication, and at most moderate alcohol consumers. Sleep characteristics were assessed using a questionnaire on habitual sleep duration, time of falling asleep, times woken up during the night, chronotype preference and the Epworth Sleepiness Scale. In general the subjects had no sleeping difficulties as they slept about 8h per night. Time to fall asleep was relatively short and times woken up during the night were low. Subject characteristics are presented in Table **1**.

**Table 1:** Subject characteristics (n=13).

Characteristic	Value
Age (y)	24.3 ± 2.5
Body weight (kg)	70.4 ± 8.6
Height (cm)	172.5 ± 6.8
Body mass index (kg/m^2^)	24.3 ± 1.7
Self-reported habitual sleep duration (h/night)	8.2 ± 1.0
Self-reported time to fall asleep	16.7 ± 4.6
Self-reported times woken up during the night	0.6 ± 0.8
Epworth Sleepiness Scale	4.5 ± 2.5
Chronotype preference (0=morning/1 = evening)	0.8 ± 0.4

All values are means ± SDs.

On the basis of respiration chamber study by Westerterp-Plantenga et al [12], power analysis with the G*Power program (version 3.1; Heinrich- Heine-Universität, Düsseldorf, Germany) showed that with an α = 0.05 and β = 0.10(power = 1 -β = 0.90), ≥11 subjects were needed. The respiration chambers ensure a highly controlled situation in which subjects are in a stable environment.

### Study design

**Figure 2:**
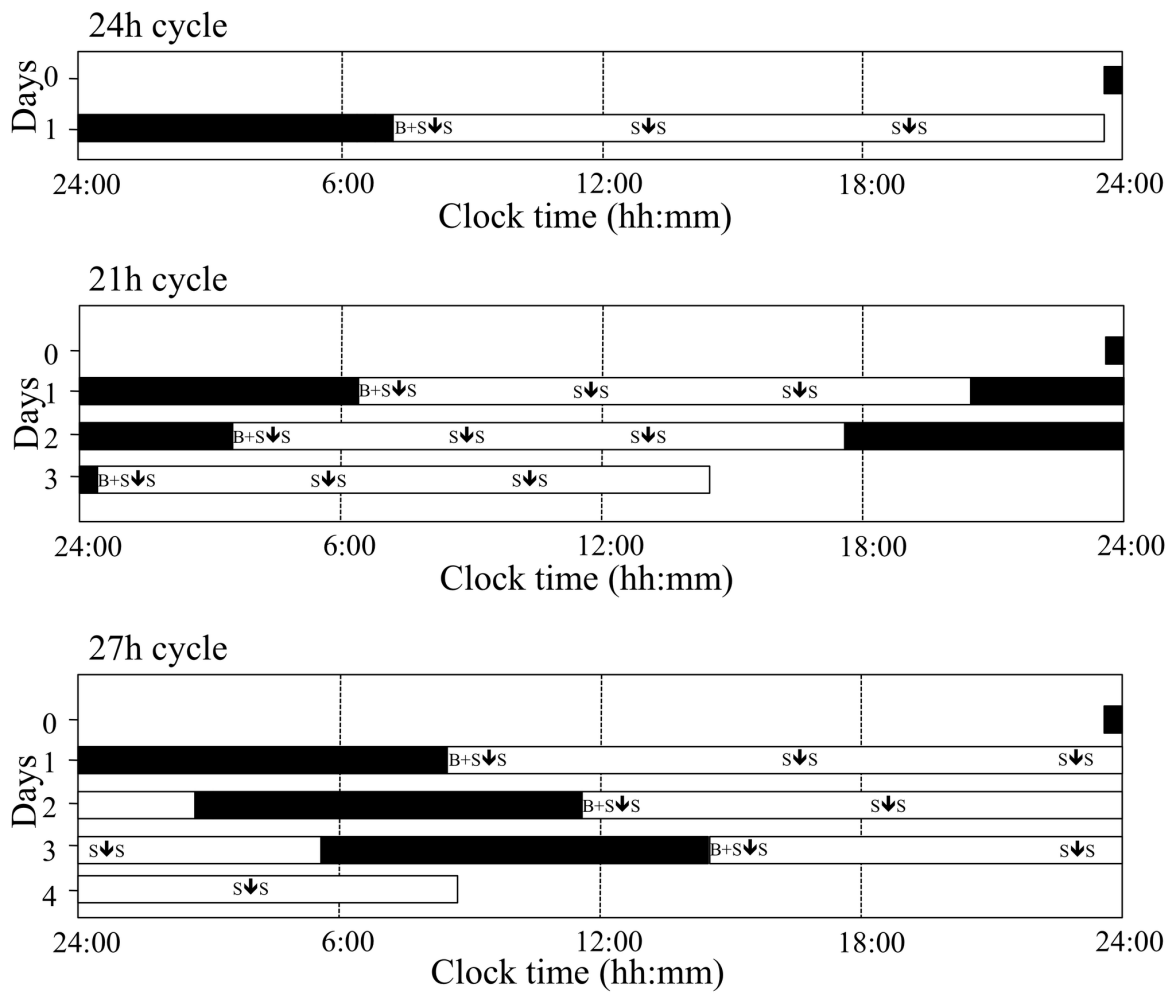
Study design. The black bars represent the sleep episodes and the white bars represent the wake episodes. Blood sampling times, saliva-sampling times are indicated as respectively B and S. ↓indicates meal times.

The study had a randomized, single-blinded, crossover design. For the 21h and 27h condition, subjects stayed in the respiration chamber during three light-entrained circadian cycles (3 x 21h or 3 x 27h). The 21h condition lasted from 2100h until 1200h three days later. The 27h condition lasted from 2100h until 0600h four days later. In the 21h cycle subjects slept 7 hours and were awake for 14 hours, while in the 27h cycle they slept 9 hours and were awake for 18 hours. Subjects underwent the 21h and 27h conditions time-blinded in random order, separated by at least four weeks. Preceding the 21h and 27h cycles, subjects stayed in the respiration chamber for 24 hours, sleeping for 8 hours and being awake for 16 hours in the chamber (Figure **2**). Subjects were confined in the chambers all the time. With the exception of partaking in strenuous exercise and sleeping, they were allowed to move freely during the wake phases. Two days before the experiment, subjects were asked to sleep according to their habitual sleep duration (8.2±1.0 hours, Table 1). During the stay in the respiration chamber polysomnography was used to monitor wake and sleep stages. Light entrainment was achieved by using daylight lamps during the waking hours (>400 lux, Energy Saver, Tornado E27, 900 lumen, Philips Lighting, Eindhoven, Netherlands) and black curtains during the sleeping hours. The endogenous melatonin rhythm, which is considered as a reliable marker of circadian timing, was used to confirm circadian misalignment. The maintenance of the 24-h melatonin secretion pattern, independent of the shifts, confirmed misalignment. This has been previously published in [10].

### Sleep monitoring

To measure wake and sleep stages, polysomnographic recordings were obtained continuously by using BrainRT (OSG BVBA, Rumst, Belgium). Before subjects entered the respiration chamber, surface electrodes for electroencephalogram (EEG), electromyogram (EMG), and electrooculogram (EOG) recording were applied according to standardized criteria [13]. All records were visually scored in 30-s epochs with standardized criteria by the same experienced person blind to the experimental condition [13]. The following sleep parameters were obtained for each subject: total sleep time (TST, consisting of all NREM+REM sleep), wake after sleep onset (WASO, min), sleep period time (defined as TST plus WASO), absolute and relative (% of sleep period time) amount of all sleep stages (Stage 1 sleep, Stage 2 sleep, SWS and REM sleep), sleep efficiency (%, defined as TST divided by time in bed), sleep latency (min, defined as the time to fall asleep) and REM sleep latency (min, defined as the time from sleep onset to the beginning of REM sleep). Daytime naps were not allowed. The sleep parameters were collected for the 24h condition and for each night of the misaligned conditions of 21h and 27h.

### Blood and saliva parameters

Every day before breakfast, fasting blood samples were collected for determination of fasting glucose and insulin concentrations (Figure 2). Furthermore, saliva samples were taken before and after each meal to determine cortisol levels (6 samples/day, Figure 2). Glucose, insulin and cortisol concentrations were determined according to a similar protocol used in previous studies conducted at the department of Human Biology, Maastricht University [14–16]. The homeostasis model assessment of insulin resistance (HOMA-IR) was calculated as follows [17]:

HOMA-IR = [fasting insulin (mU/L) x fasting glucose (mmol/L)]/22.5

### Energy intake

Two days before the experiment and during their stay in the respiration chamber subjects were fed in energy balance. The energy content of the diet was tailored individually to the energy requirements of each subject based on basic metabolic rate (BMR) calculated with the Harris and Benedict equation [18]. To estimate the total energy requirement at home, BMR was multiplied with a physical activity index (PAL) of 1.75 estimated by means of a computer simulation program [19]. The total energy requirement in the respiration chamber was estimated by multiplying the sleeping metabolic rate (SMR) of the first night with a PAL of 1.35. Energy intake was divided over the meals as 20% for breakfast, 40% for lunch, and 40% for diner. The macronutrient composition of the diet was 12/55/33 En% (protein/carbohydrate/fat). Meals were served at time-points related to cycle duration. Participants were required to finish each meal within half an hour and were not allowed to eat additional food. Water was freely available during the whole experiment.

### Statistical Analysis

Data are presented as means ± standard error of the mean (SEM), unless otherwise indicated. The area under the curve (AUC) across the day for cortisol was calculated using the trapezoidal method. ANOVA repeated measures were carried out to compare sleep parameters between the control night (24h cycle) and all nights of the 21h/27h cycle, as well as to compare sleep parameters between different nights of the 21h/27h cycle. Bonferroni corrections for multiple comparisons were applied. Linear regression analyses were completed to analyze the inter-individual relationship between different sleep stages and blood/saliva parameters. All tests were two-sided and differences at P < 0.05 were considered significant. Data were analyzed using SPSS version 18.0 for Macintosh, OS X (SPSS Inc., Chicago, IL, USA).

## Results

### Effects of sleep curtailment/extension

The control night (24h cycle), the first night of the 21h cycle and the first night of the 27h cycle started at 23h30 and differed only in the duration of sleep opportunity. As a consequence of this design, the effect of sleep duration on sleep architecture, independent of the circadian rhythm, could be determined by comparing the first night of the 21h and 27h cycle to the control night. Subjects slept on average 375.5±8.9min during the 21h cycle and 490.2±7.0min during the 27h cycle compared to 438.8±6.0min during the 24h cycle. Shorter sleep duration, independent of a circadian shift, resulted in a significant decrease of REM sleep, a significant decrease of stage 2 sleep and preservation of SWS. Longer sleep duration, independent of a circadian shift, resulted in a significant increase of REM sleep, stage 2 sleep and SWS (Table **2**).

**Table 2:** Absolute (min) and relative (%) duration of sleep parameters during the 21h cycle and the 27h cycle compared to the control night (24h cycle). All values are means ± SEMs (n=13).

Parameters	24h night	21h 1st night	21h 2^nd^ night	21h 3rd night	27h 1^st^ night	27h 2^nd^ night	27h 3^rd^ night
WASO (min)	9.6±3.2	20.5±8.0	11.0±5.1	34.3±6.3*^, c^	17.0±4.9	24.3±7.3	18.0±1.9*
(%SPT)	2.1±0.7	5.2±2.0	2.8±1.3	8.6±1.5*^, c^	3.4±1.0	4.6±1.4	3.5±0.4
TST (min)	438.8±6.0	375.5±8.9*	389.1±6.8*	365.4±10.2*	490.2±7.0*	501.4±8.0*	502.7±3.7*
Stage 1 (min)	5.0±1.5	6.8±2.2	6.5±1.9	6.5±2.2	5.4±1.8	7.8±2.4	6.2±1.7
(%SPT)	1.1±0.3	1.7±0.6	1.7±0.5	1.6±0.4	1.1±0.4	1.5±0.4	1.2±0.3
Stage 2 (min)	214.9±10.5	177.2±9.8*	194.3±6.8*	174.3±8.3*	244.8±11.1*	234.6±7.7	220.8±4.4^b^
(%SPT)	48.0±2.3	44.7±2.4	48.6±1.7	43.4±1.6^c^	48.3±2.1	44.6±1.3	42.6±0.9*^, b^
SWS (min)	125.7±13.1	125.4±13.5	108.9±9.2^a^	108.2±7.6^b^	131.9±14.2*	125.3±11.6	136.8±9.7
(%SPT)	27.4±2.9	31.7±3.5	27.2±2.2^a^	27.0±1.8^b^	25.3±2.8	23.9±2.2*	26.2±1.8
REM sleep (min)	93.1±8.2	66.1±5.4*	79.3±4.9^a^	76.3±4.7	108.1±7.6*	133.7±9.5*^, a^	138.9±8.5*^, b^
1^st^ part of the night	22.5±5.2	19.9±4.1	33.0±2.6^a^	31.9±6.7	32.5±5.6*	46.2±5.3*^, a^	62.2±5.3*^, b, c^
2^nd^ part of the night	70.6±7.6	46.2±3.7*	46.3±4.4*	44.4±4.6*	75.7±7.1	87.5±7.4	76.7±5.8
(%SPT)	20.6±1.7	16.7±1.3*	19.8±1.2^a^	19.3±1.4	21.2±1.4	25.4±1.8*^, a^	26.7±1.6*^, b^
Sleep latency (min)	31.4±5.7	23.0±4.0	16.9±3.1	9.5±1.3*^, b, c^	31.4±5.7	12.7±2.4*^, a^	8.5±2.1*^, b, c^
REM sleep latency (min)	114.3±15.5	110.1±15.5	65.0±9.5*^, a^	82.2±15.4	114.3±15.5	83.7±11.8*^, a^	93.1±13.8
Sleep efficiency (%)	91.4±1.2	89.4±2.1	92.6±1.6	87.0±2.4	90.8±1.3	92.8±1.5	93.1±0.7

WASO: Wake After Sleep Onset; TST: Total Sleep Time; SWS: slow-wave sleep; REM: rapid eye movement. * Differences to control night. ^a^ Within-cycle differences between 1st and 2nd night. ^b^ Within-cycle differences between 1st and 3rd night. ^c^ Within-cycle differences between 2nd and 3rd night.

### Intra-individual effects of shifted sleep on sleep architecture

A phase advance resulted in a significantly increased time awake (WASO) during the third night compared to the second night. Moreover, during the third night of the phase advance sleep latency was significantly decreased compared to the first and second night. Stage 2 sleep stayed significantly decreased during all three nights compared to the control night. SWS decreased significantly during the second night and stayed decreased during the third night compared to the first night. Although REM sleep during the first night was significantly decreased compared to the control night, REM sleep showed a rebound during the second and third night (Table 2).

A phase delay resulted in a significant increased time awake (WASO) during the third night compared to the control night. Moreover, during the third night of the phase delay sleep latency was significantly decreased compared to the first and second night. Stage 2 sleep significantly decreased again during the third night. Although SWS during the first night was significantly increased compared to the control night, there was no significant difference in SWS between the three nights of the 27h cycle. REM sleep further increased significantly during the second and third night (Table 2).

A different role of REM sleep in the first part of the night compared to the latter part of the night has been observed previously [20]. Therefore, we further investigated the distribution of REM sleep over the night in relation to circadian misalignment (Table 2). Subjects’ time in bed was respectively 7, 8 or 9 hours, so REM sleep during the first part of the night is calculated as REM sleep during the first respectively 3.5, 4 or 4.5 hours of time in bed. A phase advance resulted in a significant decrease in REM sleep in the second part of the night (calculated as REM sleep during the last 3.5 hours of time in bed) compared to the control night. The REM rebound in the second night is observed as a significantly increased REM sleep in the first part of the night. Consequently, a phase advance resulted in significantly decreased REM sleep latency.

A phase delay resulted in a significant increase in REM sleep in the first part of the night compared to the control night. The further increase in REM sleep in the second and third night is observed as a significantly increased REM sleep in the first part of the night. Consequently, a phase delay resulted in significantly decreased REM sleep latency. Thus, a phase advance as well as a phase delay resulted in a change in distribution of REM sleep over the night (Table 2).

### Inter-individual relationships between sleep architecture, cortisol levels and HOMA-IR index

**Figure 3:**
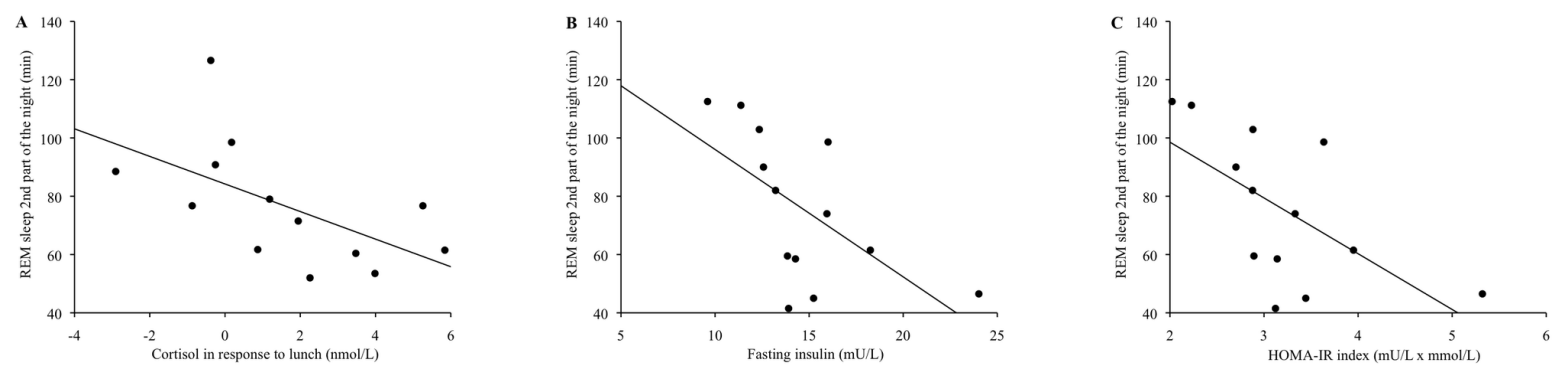
Relationship between REM sleep during the 2^nd^ part of the night and cortisol concentrations in response to lunch (nmol/L) during phase delay. R^2^=0.334, P<0.05, n=13 (A). Relationship between REM sleep during the 2^nd^ part of the night and fasting insulin concentrations (mU/L) during phase delay. R^2^=0.374, P<0.05, n=13 (B). Relationship between REM sleep during the 2^nd^ part of the night and HOMA-IR index (mU/L x mmol/L) during phase delay. R^2^=0.382, P<0.05, n=13 (C).

Possible changes in cortisol concentrations, as an indicator of HPA-axis activity were observed as follows. Linear regression analyses showed that during phase delay cortisol concentrations across the day were inversely related to the amount of REM sleep (R^2^=0.328, P=0.041). More specifically, the cortisol concentrations in response to lunch were inversely related to the total amount of REM sleep (R^2^=0.542, P=0.004) and to the amount of REM sleep during the second part of the night (R^2^=0.334, P=0.021, Figure 3A). This suggests that subjects with less total REM sleep during the second part of the night had higher cortisol concentrations.

Possible changes in fasting insulin concentrations and HOMA-IR index, as indicators of insulin sensitivity, were observed as follows. During phase delay, shorter TST was associated with higher fasting insulin levels (R^2^=0.575, P=0.003) and a higher HOMA-IR index (R^2^=0.49, P=0.008). In addition, shorter REM sleep was associated with higher fasting insulin levels (R^2^=0.375, P=0.026) and a higher HOMA-IR index (R^2^=0.363, P=0.029). Particularly the amount of REM sleep during the second part of the night was inversely related to fasting insulin levels (R^2^=0.374, P=0.026, Figure 3B) and to the HOMA-IR index (R^2^=0.382, P=0.024, Figure 3C). This suggests that subjects with less total REM sleep during the second part of the night had higher fasting insulin levels and a higher HOMA-IR index.

In the control and the phase-advanced condition, no relations were found for cortisol, fasting insulin concentrations and HOMA-IR index with sleep parameters.

## Discussion

In the present study, the intra-individual effects of circadian misalignment on sleep architecture were examined. Moreover, inter-individual relationships between different sleep stages and cortisol concentrations, as an indicator of HPA-axis activity, and the fasted HOMA-IR index, as an indicator of insulin sensitivity, were investigated.

Intra-individual analyses showed that circadian misalignment, both a phase advance and a phase delay of the sleep period, resulted in disruption of the normal phase distribution between SWS and REM sleep in that REM sleep was relatively phase advanced to SWS and sleep onset. This abnormal circadian sequencing results in shortening of REM sleep latency. This short latency to REM sleep is typical of narcoleptic and depressive patients [21]. In addition to the change in distribution of REM sleep over the night, a phase advance resulted in increased time awake after sleep onset, which is in agreement with the study of Wyatt et al, showing a significant effect of circadian phase on time awake after sleep onset [22]. Consequently, phase advancing the time to go to bed results in diminished sleep continuity. In addition, a phase advance resulted in decreased SWS, which is consistent with the dominant homeostatic modulation of SWS found by Wyatt et al. [22]. During the 21h cycle, subjects are awake for 14h compared to 16h during a 24h day. It may be that during this 14h less homeostatic sleep pressure is built up before sleep, which may explain the reduced amount of SWS during the phase advance. Furthermore, advancing the sleep period phase resulted in a REM sleep rebound since REM sleep was decreased due to decreased time in bed (comparison 7h vs. 8h time in bed). During the second night of the phase advance shift REM sleep latency significantly decreased. This decrease in REM sleep latency may be caused by the REM sleep rebound following REM sleep deprivation [23]. Similarly to the phase advance, the phase delay resulted in increased time awake during the night resulting in decreased sleep continuity. Moreover, phase delaying the time to go to bed significantly increased REM sleep with a decreased REM sleep latency, which is consistent with the pronounced circadian modulation of REM sleep found by Dijk et al. [24]. REM sleep is normally concentrated in the second half of the night due to a circadian disposition for REM sleep to occur at this particular time of the day [25]. Consequently, delaying the phase of full-length sleep period results in a higher percentage of REM sleep compared to advancing the phase of a full-length sleep period.

Inter-individual analyses showed that subjects with relatively less REM sleep, particularly during the second part of the night, showed higher cortisol concentrations and a higher HOMA-IR index. In concordance with a study by Knutson et al. who found that shorter sleep duration measured using wrist actigraphy was associated with higher fasting insulin levels and a higher HOMA-IR index [26], our study indicates a negative correlation between total sleeping time and fasting insulin concentrations and between total sleeping time and the HOMA-IR index. In addition, shorter REM sleep especially during the second part of the night was associated with higher fasting insulin concentrations and higher HOMA-IR index. Koren et al. found positive associations between SWS duration and insulin secretory measures in obese adolescents [27], while in the present study no correlations between SWS and the HOMA-IR index were observed. Koren et al. studied individuals with a mean age of 14.4 years, while in the present study the mean age was 24.3 years, this may explain the discrepancy. Moreover, the discrepancy may be explained by the differences in sleep architecture between obese and non-obese subjects. Obese adolescents show an abnormal sleep pattern with above average SWS but below normal REM sleep percentage [28]. The association between REM sleep and cortisol levels is in accordance with the results described by Van Cauter et al., who observed an inverse relationship between nadir cortisol levels and REM sleep [29]. It is striking that especially REM sleep during the second part of the night was negatively associated with cortisol levels. Wu et al. already showed that sleep at the 03:00-06:00 period, during the circadian nadir, is important in protecting normal physiological rhythms and function of the HPA-axis [30].

The circadian system, however, does not only causes circadian modulation of sleep stages in particular REM sleep, but in parallel it also causes modulation of fasting glucose, insulin and cortisol concentrations [10]. In previous work, phase advancing the sleep period resulted in an increased insulin response to food intake and glucose secretion increased when the sleep period was delayed [10]. Moreover, circadian misalignment caused flattening of the cortisol-secretion pattern [10]. Therefore, the reported inter-individual correlations may also be explained by parallel changes in the circadian phases of REM sleep, fasting insulin and cortisol concentrations, which are all under circadian control.

Finally, the present study showed the effects of sleep duration on sleep architecture. Shortening sleep duration to 7h of time in bed decreased the amount of stage 2 and REM sleep, while SWS was preserved compared to the control night of 8h of sleep. These findings correspond with other studies investigating sleep restriction to only 4h or 5.5h time in bed [31–34]. When the total time in bed is reduced, the sleep system responds by primarily decreasing stage 2 sleep and REM sleep while SWS does not seem to be affected. The preservation of SWS during sleep restriction supports the presumption that SWS is the most restorative part of sleep [25]. With respect to our results on longer sleep duration, the absolute amount of stage 2 sleep, SWS and REM sleep was increased but the percentage of the sleep period spent in these stages remained constant.

Important to note is that all significant findings in this study were observed when considering the absolute values. Despite the difference in time in bed between conditions, it is crucial to compare the absolute duration of sleep stages and not the relative duration; in that case percentages of sleep stages would seem higher as the denominator is reduced sleep time, while absolutely it has not increased at all.

In the present study, subjects are submitted to a sudden advance or delay in their dark-light, rest-activity, sleep-wake cycle, similar to what occurs in jet lag or shift work rotations. Such a protocol allows for the effects of circadian modulation to be observed in the absence of sleep and for the effects of sleep to be observed at an unusual circadian time. In contrast to a forced desynchrony protocol, we were not able to distinguish between the independent effects of the circadian system and behavioral cycles. Possible limitations of the present study are the lack of an adaptation night and that the 24h condition each time took place before the 21h and 27h conditions. However, there were no significant differences in sleep efficiency between different nights diminishing the possible sequence effect (Table 2).

Taken together, intra-individually we found a REM sleep rebound during the phase advance and increased REM sleep during the phase delay moreover REM sleep distribution over the night changed with a relatively shorter REM sleep duration during the second part of the night. Inter-individually we found that shorter REM sleep duration during the second part of the night was correlated with higher cortisol concentrations and a higher HOMA-IR index. This may also explain why not all people are sensitive for circadian misalignment and its negative metabolic consequences.

In conclusion, circadian misalignment, both a phase advance and a phase delay, resulted in a significant change in sleep architecture, especially a shift in REM sleep. Inter-individually, shorter REM sleep during the second part of the night was associated with dysregulation of the HPA-axis, as indicated by increased cortisol concentrations, and reduced insulin sensitivity. Dysregulation of the HPA-axis and insulin resistance are hallmarks of several metabolic diseases such as type-2 diabetes and obesity, which are known to be associated with circadian misalignment.

## Supporting Information

Protocol S1 Trial Protocol.
(PDF)

Checklist S1 CONSORT Checklist.
(DOC)
