# Supplementary material for: Sleep Architecture When Sleeping at an Unusual Circadian Time and Associations with Insulin Sensitivity
Source: PLoS One. 2013 Aug 8;8(8):e72877. doi: 10.1371/journal.pone.0072877 (PMC3738551; doi:10.1371/journal.pone.0072877)
Supplement: Protocol S1 — Trial Protocol. (PDF) [file pone.0072877.s001.pdf]

- Research proposal -

F Rutters, H Gonnissen, R Hursel, MS Westerterp-Plantenga

April 2010

Protocol title

Effects of circadian misalignment and sleep disturbance on feeding patterns, obesity and the metabolic syndrome

Protocol ID NL 31568

Short title Effects of circadian misalignment

Version Second

Date 12/04/2010

Coordinating investigator Prof. M.S. Westerterp-Plantenga; Maastricht University; m.westerterp@hb.unimaas.nl; 043-3881566

Principle investigator dr. F. Rutters: Maastricht University; f.rutters@hb.unimaas.nl; 043-3882123

Sponsor none

Independent physician dr. H. Kuipers; MUMC+; 043-3881493

Laboratory sites Maastricht University

Protocol Signature Sheet

Head of department Prof. R. Mensink

Coordinating investigator Prof. M.S. Westerterp-Plantenga

Table of contents

Protocol title 2

List of abbreviations and relevant definitions 4

Summary 5

1. Introduction and rationale 7

2. Primary objectives 10

***3.* Study design** 11

4. Study population 13

5. Treatment of subjects 15

7. Methods 16

8. Safety reporting 26

9. Statistical analysis 28

10. Ethical considerations 29

11. Administrative aspects and publication 32

12. References 34

List of abbreviations and relevant definitions

BMI: Body Mass Index

SCN: suprachiasmatic nucleus (brain structure)

AEE: activity induced energy expenditure

EEG: electroencephalogram

ECG: Electrocardiography

EMG: Electromyography

EOG: Electrooculography

REM: Rapid eye movement sleep

VAS: visual analogue scales

POMS: Profile of Mood States

STAI-state: State Trait Anxiety Index

TFEQ: Three Factor Eating Questionnaire

TEE: total energy expenditure

SMR: sleeping metabolic rate

DEE: diet-induced energy expenditure

RQ: respiratory quotient

RMR: Resting Metabolic Rate (consisting of SMR and DEE)

PAL: Physical Activity Level

BMR: Basal Metabolic Rate

Summary

Rationale: The prevalence of obesity has increased worldwide to epidemic proportions. Until now, recommendations for improving food choice, food intake patterns, and energy intake, as well as for normalizing physical activity, have not resulted in solutions for prevention and treatment of obesity. It may be that advices on the energy balance components energy intake and energy expenditure lack the interaction with the subjects' individual circadian alignment, or that circadian alignment is disturbed, due to environmental pressure.

Objective: To determine the consequences of circadian misalignment for energy intake and energy expenditure, as well as sleep quality, mood, feelings of hunger and satiety, food choice, and the reward system.

Study design: Subjects will stay time blinded in the respiration chamber to measure energy intake and energy expenditure, as well as sleep quality, mood, feelings of hunger and satiety, food choice, and the reward system. During their stay in the chamber, subjects will be fed in energy balance in a food-entrained way, at time-points related to their cycle duration. EEG will be used to measure wake and sleep phases continuously, in order to determine sleep quality and sleep time. Heart rate will be monitored using a ECG and body temperature using a CorTemp™ Data Recorder. Appetite and mood profiles will be measured hourly and before and after each meal, by anchored 100 mm visual analogue scales and by POMS and STAI-state questionnaires. Also, blood-parameters will be measured before and after a meal. Finally, effects on food reward and energy intake will be measured by means of a validated wanting and liking computer-test, before and during the evening meal before subjects leave the chamber. This evening meal consists of subjects' food choice, and will be given *ad libitum.* Food choice, rewarding characteristics, macronutrient composition, energy-density, and energy content will be calculated.

Study population: 16 male subjects in good health, weight stable, non-smokers, not using medication, not having any sleep problems, and at most moderate alcohol users. They have to be overweight, BMI 25-29 kg/m^{2,} adolescent and young adult, age 18-30 yrs, men, who are evening people.

Intervention: Subjects stay for each condition, for 3 circadian cycles (3 x 21 h or 3 x 27 h), in the absence of time cues, in a controlled situation under controlled energy balance conditions in a respiration-chamber.

Main study parameters/endpoints: energy expenditure and activity induced energy expenditure (AEE), substrate oxidation, sleep quality, as well as mood, feelings of hunger and satiety, food choice and energy intake, the rewarding value of food (wanting and liking), endocrinological aspects, and body temperature

Nature and extent of burden: This study does not include any major risks for the subjects. During the time-blinded period two researchers will always be present during the subjects stay in the respiration chamber. There are no risks for the subjects in using any of the meals because people with certain food allergies are excluded and all products are regular food items available at the local supermarket (AH). The "thermometer pill" that they have to swallow is completely harmless and runs through the digestive system, where it eventually will exit the body via the feces. Blood sampling in this study is limited and without side effects, apart from its usual risks of minor bruising. Urine sampling is done in urine bottles with 10 ml of diluted HCl, which might pose a risk for the subjects, however they will be carefully instructed on how to sample the urine and handle the urine bottles. The subject needs to follow the prescribed schedule on sleeping, filling in questionnaires and eating. The experiment will take about 144 hours of the subject's time.

Benefit: This study does not have any benefits for the subjects themselves, but will give possible new knowledge for treatment of obesity.

1. Introduction and rationale

The prevalence of obesity has increased worldwide to epidemic proportions (1). In the period 1993 to 1997, 40.2% men and 25.0% women of the Dutch population (age 37-43y) were regarded as overweight (Body Mass Index: (BMI) 25-29.9 kg/m^2), and 8.5% men and 9.3% women were regarded as obese (BMI>30 kg/m^2). This is considered as a major health problem, since obesity is associated with an increased risk for a number of serious diseases, such as coronary heart diseases, hypertension, non-insulin dependent diabetes, pulmonary dysfunction, and certain types of cancer (1). Developing strategies for weight loss as well as trying to reveal the causes of obesity is important, because even a modest weight loss (5-10% of initial body weight) markedly reduces the risk for mortality and morbidity.

Evidence from interventions to reduce body-weight is very limited (2, 3). Current strategies for weight reduction include dietetic interventions often combined with behaviour therapy, although the effect is controversial. Meta-analyses show that dietary treatment achieves only temporary and short-term weight loss (2, 3). Multidisciplinary treatment programs report improvements in body composition, fitness and modest short-term weight loss. For long-term treatment success permanent lifestyle changes are necessary with regard to energy balance, food intake, implying physical activity patterns and behavior to emotional stress. Until now, recommendations for improving food choice, food intake patterns, and energy intake, as well as for normalizing physical activity, have not resulted in solutions for prevention and treatment of obesity. It may be that advices on the energy balance components energy intake and energy expenditure lack the interaction with the subjects' individual circadian alignment, or that circadian alignment is disturbed, due to environmental pressure.

Circadian rhythm is an essential component of energy homeostasis, as it represents an organism's ability to coordinate daily patterns in activity, feeding, energy utilization and energy storage across the daily 24-h cycle (4, 5). Recent studies in animals and humans have demonstrated that even mild disruptions of circadian regulation and sleep can have profound effects on metabolism regulation and obesity (6-8).

For example, the suprachiasmatic nucleus (SCN) of the hypothalamus has been studied, which is considered to be the master circadian clock that orchestrates and synchronizes behavioural and physiological rhythms under normal circumstances. Metabolic processes are easily decoupled from the primarily light-driven SCN, when food intake is desynchronized from normal diurnal patterns of activity, thereby demonstrating that the circadian system is responsive to changes in energy supply and metabolic status (8). Furthermore, it has been shown that coupling of feeding schedules with caloric restriction shifts behavioural and physiological circadian rhythms and gene expression in the SCN to meal-time in an advanced direction, probably due to increased hunger, whereas high-fat intake shifts circadian rhythms in a delayed direction with especially a high proportion of energy intake late in the evening (9). This close interaction is likely to be critical for normal circadian regulation of metabolism, and may also, together with directly related possible sleep disturbance, underlie the disruption of proper metabolic rhythms observed in metabolic disorders, such as obesity and type-II diabetes

Until now, only one study has assessed the consequences of circadian misalignment in humans. Scheer et al 2009 (10) subjected ten adults to a 10 day protocol, wherein subjects ate and slept at all phases of the circadian cycle-achieved by scheduling a recurring 28h day. Circadian misalignment, when subjects ate and slept approximately 12 h out of phase from their habitual times, systematically decreased leptin levels, and increased glucose concentrations despite increased insulin levels. It also completely reversed the daily cortisol rhythm, increased mean arterial pressure, and reduced sleep efficiency. Notably, circadian misalignment caused 3 of 8 subjects (with sufficient available data) to exhibit postprandial glucose responses in the range typical of a prediabetic state. Circadian misalignment appeared to result in metabolic and cardiovascular changes as seen in obese subjects, and therefore present to be a risk for overweight and obesity. The study by Scheer et al (10) only studied the metabolic and cardiovascular consequences, however circadian misalignment may also affect other parts of the energy balance, namely energy intake and energy expenditure (11). Both sides of the energy balance are regulated by the circadian rhythm (11). Circadian changes in energy expenditure have been shown in resting metabolic rate, which is about 6% higher in the afternoon than in the morning (12).

Also, circadian changes in energy intake have been shown, as reverse feeding of nocturnal active animals during the light phase can result in increased weight gain (13). Therefore, the aim of the present study is first to determine the consequences of circadian misalignment for energy intake and energy expenditure, sleep quality, mood, feelings of hunger and satiety, food choice, and the reward system. We hypothesize that circadian misalignment may result in a disturbed energy balance, as a consequence of decreased energy expenditure and increased energy intake. Furthermore, it may alter substrate oxidation, decrease sleep quality, increase feelings of hunger and decrease feelings of satiety, alter food choice towards more high fat and sugary food, and alter the rewarding value of food towards higher wanting and decreased liking of foods. All these changes have previously been shown in obese subjects and therefore may illustrate a pre-obese state in the overweight subjects we will be studying, which when circadian misalignment is prolonged, may result in overweight and obesity.

2. Primary objectives

The primary objectives of the study are to assess the effect of circadian misalignment on:

- energy expenditure and activity induced energy expenditure (AEE)
- substrate oxidation
- sleep quality and sleep time
- mood, feelings of hunger and satiety
- food choice and energy intake
- the rewarding value of food (wanting and liking)
- relevant endocrinological parameters
- body temperature

***3.* Study design**

Study design

Subjects will stay time blinded in the respiration chamber to measure energy intake and energy expenditure, as well as sleep quality, mood, feelings of hunger and satiety, food choice, and the reward system (14), during three light-entrained circadian cycles of a 1:2 ratio. A short cycle will be 21 hrs (7/14), a long cycle 27 hrs (9/18). In contrast to the study by Scheer et al. (10) in which a phase difference of 24 hours was induced, we will induce a maximal phase difference of 9 hours to minimize the duration of the experiment and the strain on the subjects. The chosen duration of 3 short or long circadian cycles is a compromise between a minimum duration in order to be able to obtain a clear result, and a maximum duration that still is bearable for the subjects. The chosen duration is also based upon experience with respiration chamber studies lasting for 84 h (15, 16). The total duration of the experiment is 81 + 63 = 144 h (6 days), with at least 1 month in between the long and/or short cycles, to avoid overlap and to make sure the subjects can get back to their regular circadian rhythm before they are subjected to the altered cycles. Light-entrainment will be achieved using day-light lamps during the waking hours and black curtains during the sleeping hours. During food-entrainment, light conditions will be dim light continuously, (about 1.8 lux) (10), to minimize any light-entrained influence on the circadian system. During their stay in the chamber, subjects will be fed in energy balance (17) in a food-entrained way, at time-points related to their cycle duration. EEG will be used to measure wake and sleep phases continuously, in order to determine sleep quality. Heart rate will be monitored using a ECG and body temperature (18) using a CorTemp™ Data Recorder (HQInc., Palmetto (FL), USA). Appetite and mood profiles will be measured hourly and before and after each meal, by anchored 100 mm visual analogue scales (19) and by POMS and STAI-state questionnaires (20, 21). Also, blood-parameters will be measured before and after a meal (6, 7). Finally, effects on food reward and energy intake will be measured by means of a validated wanting and liking computer-test (22), before and during the evening meal before subjects leave the chamber. This evening meal consists of subjects' food choice, and will be given *ad libitum.* Food choice, rewarding characteristics, macronutrient composition, energy-density, and energy content will be calculated.

The experimental design of the study is presented in **figure 1** by a raster plot.

Figure 1

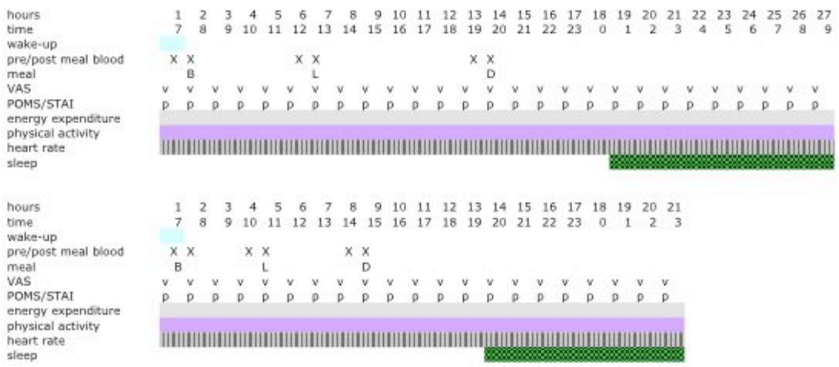

4. Study population

1. Population

In total 16 male subjects from the student population will be recruited by means of advertisements on notice boards at Maastricht University. Men will be recruited as in women oral contraceptive usage and phase of the menstrual cycle have to be taken into account; in addition the expected differences are that large that we would require twice the number of subjects. We will recruit adolescent and young adult men (age 18-30 yrs), who are overweight (BMI 25-29 kg/m^2), as they are thought to be susceptible for development of obesity. The men also have to be evening people, with a naturally long circadian cycle and with no sleep problems (such as insomnia, daytime hypersomnia, or repeated waking up during the night), which will be assessed using the validated Munich Chronotype Questionnaire (23). The subjects have to be in good health, non-smokers, not using medication, no food allergies, weight stable and at most moderate alcohol users. This will be evaluated using questionnaires on health, use of medication, smoking behaviour, food allergies and alcohol consumption. During screening body-weight, height, waist circumference, and hip circumference will be measured. No additional physical tests will be performed to evaluate the inclusion criteria; the investigator will evaluate reliability of the subject during the screening. Furthermore, habitual physical activity will be estimated with the Baecke questionnaire (24), which consists of three components: work activity, sports activity and leisure activity. The Dutch translation of the Three Factor Eating Questionnaire (TFEQ) (25) will be used to determine aspects of eating behaviour: cognitively dietary restraint; disinhibition/emotional eating; and general hunger. A list of food products that will be used during the experiment will be presented to the subject. If a subject does not like any of the items, they will be replaced, to eliminate the effect of food preferences on feelings of hunger and satiety.

2.Inclusion criteria

Subjects have to be in good health, weight stable, non-smokers, not using medication, not have any sleep problems, and at most moderate alcohol users. They have to be overweight, BMI 25-29 kg/$m^{2,}$ adolescent and young adult, age 18-30 yrs, men, who are evening people.

3. Exclusion criteria

Subjects will be excluded if they are not in good health, changed weight (3 kg) in the last 3 months, using medication, smokers, alcohol users and/or have sleep problems (such as insomnia, daytime hypersomnia or repeated waking up during the night). Also if their BMI is under 25 or over 29 $kg/m^{2,}$ or aged under 18 or over 30 yrs, or if they are morning people; the expected differences may be that large that we would require twice the number of subjects.

4. Sample size

The primary parameters/endpoints are the changes in the energy expenditure and energy intake. The study by Scheer et al. (10) did not study 24h energy expenditure or total energy intake. No other studies were found, observing the effect of circadian misalignment on 24 h energy expenditure or total energy intake. To do a sample size calculation a study on sleep deprivation was used. The study by Brondel et al. (26) showed an increase in energy intake after 4 h sleep compared to 8 h sleep in healthy young men (3037±853 vs. 2478±512 Kcal, $p<0.01$). With an α of 0.05 and β of 0.10 (power=1-β=0.90) the number of subjects needed is:

$$N=10x(SD)^2/(mean1-mean2)^2+0.96$$
$$N=10x(512)^2/(3037-2478)^2+0.96$$
$$= 9 \text{ subjects needed}$$

Taking drop out and a smaller phase shift (1 night with 4 hours sleep deprivation compared to 3 days of 3 hour phase shift) into account, 16 subjects will be included in the experiment. 16 subjects will also be sufficient to be able to find significant differences in energy expenditure, appetite profiles and endocrinological parameters, based upon the studies by Lejeune et al. and Veldhorst et al (27, 28).

5. Treatment of subjects

1. Investigational product/treatment

The aim of the present study is first to determine the consequences of circadian misalignment for energy intake and energy expenditure, as well as mood, feelings of hunger and satiety, food choice, and the rewarding value of food. Subjects stay for each condition, for 3 circadian cycles (3 x 21 h or 3 x 27 h), in the absence of time cues, in a controlled situation under controlled energy balance conditions in a respiration-chamber. A short cycle will be 21 hrs (7/14), which represents the cycle of morning people, and a long cycle will be 27 hrs (9/18), which represents the cycle of evening people. In order to identify effects of circadian misalignment, subjects undergo randomly the short or long circadian cycles.

7. Methods

1. Study parameters/endpoints

1.1 Main study parameters/endpoints

- **energy expenditure**, which will be measured continuously during the 21h or 27h cycles via oxygen consumption and carbon dioxide production that will be measured in the respiration chamber. The concentrations of oxygen and carbon dioxide will be measured using a paramagnetic O_2 analyzer and an infrared CO_2 analyzer.
- **food choice** and **energy intake**, which will be measured before and after diner on the last day of the 3 circadian cycles via a wanting and liking test on a computer (22). During the rest of the stay the subjects have to be fed in energy balance.

We hypothesize that circadian misalignment may result in a disturbed energy balance, as a consequence of decreased energy expenditure and increased energy intake, as it alters food choice towards more high fat and sugary food.

1.2 Secondary study parameters/endpoints

- **activity induced energy expenditure (AEE)**, which will be measured continuously during the 21h or 27h cycles via a triaxial accelerometer (Tracmor, Maastricht, The Netherlands) on a belt.
- **substrate oxidation**; carbohydrate, fat, and protein oxidation will be calculated using O_2 consumption and CO_2 production and urinary nitrogen excretion, with the formula of Brouwer (29). Urine will be collected every 21 h or 27h. Volume and nitrogen concentration will be measured, the latter using a nitrogen analyzer.
- **sleep quality and sleep duration**, which will be measured continuously via EEG measurements. Sleep quality represents the amount of minutes in which the subject is either in sleep phase III, IV plus Rapid eye movement sleep (REM) divided by total duration of sleep. Sleep will be registered using 6 electrodes on the head to measure brain activity (EEG), 2 electrodes on the chin to measure muscle activity (EMG), 2 electrodes around the eyes to measure eye movement (EOG) and 2 electrodes on the chest to measure heart rate and cardiac function (ECG).

- **mood, feelings of hunger and satiety**, will be measured hourly and before and after each meal, by visual analogue scales (VAS) (19) and by Profile of Mood States (POMS) and State Trait Anxiety Index (STAI) questionnaires (20, 21).
- **the rewarding value of food (wanting and liking)**, will be measured before and after diner on the last day of the 3 circadian cycles via a wanting and liking test on a computer (22).
- **endocrinological parameters**, will be measured before and after a meal via a catheter inserted into an ante-cubital vein of the contra-lateral arm. From the blood samples the following parameters will be determined melatonin, leptin, glucose, insulin, ghrelin, and GLP-1.
- **body temperature,** which will be measured continuously via a CorTemp™ Data Recorder (HQInc., Palmetto (FL), USA). Subjects will swallow a "thermometer pill" (CorTemp™ Ingestible Core Body Temperature Sensor) that wirelessly transmits core body temperature as it travels through the digestive tract.

We hypothesize that circadian misalignment may alter substrate oxidation, decrease sleep quality, increase feelings of hunger and decrease feelings of satiety, and alter the reward system towards higher wanting and decreased liking of foods. All these changes have previously been shown in obese subjects and therefore may illustrate a pre-obese state in the overweight subjects that we will study, which when circadian misalignment is prolonged, may result in overweight and obesity.

2. Randomisation, blinding and treatment allocation

Partly randomized single-blinded cross-over design.

3. Study procedures

Energy expenditure and activity induced energy expenditure (AEE)

Oxygen consumption and carbon dioxide production will be measured in the respiration chamber (14). The respiration chamber is a 14 m^3 room, furnished with a bed, chair, computer, television, radio, dvd-player, telephone, intercom, sink and toilet. The room is ventilated with fresh air at a rate of 70-80 l/min. The ventilation rate is measured with a dry gas meter. The concentrations of oxygen and carbon dioxide will be measured using a paramagnetic O_2 analyzer and an infrared CO_2 analyzer. During each 15-min period six samples of outgoing air for each chamber,

and one sample of fresh air, zero gas, and calibration gas will be measured. The gas samples to be measured will be selected by a computer that also stores and processes the data (14).

21 h or 27h energy expenditure consists of sleeping metabolic rate (SMR), diet-induced energy expenditure (DEE), and AEE. 21 h or 27h energy expenditure and 21 h or 27h respiratory quotient (RQ) will be calculated using the formula of Brouwer (29). Physical activity is monitored using a radar system working on the Doppler principle. The subjects wear a triaxial accelerometer (Tracmor, Maastricht, The Netherlands) on a belt during the entire stay in the chamber, to measure physical activity as well (30). SMR is defined as the lowest mean energy expenditure measured over three consecutive hours when the subject is asleep. DEE will be calculated by plotting energy expenditure against radar output, both averaged over 30-min periods (31). The intercept of the regression line at the lowest radar output represents the energy expenditure in the inactive state (Resting Metabolic Rate; RMR), consisting of SMR and DEE. DEE will be determined by subtracting SMR from RMR. Activity-induced energy expenditure will be determined by subtracting RMR from 21 h or 27h energy expenditure. The Physical Activity Level (PAL) will be calculated by dividing 21 h or 27h energy expenditure by SMR.

<u>Substrate oxidation</u>

Carbohydrate, fat, and protein oxidation will be calculated using O_2 consumption and CO_2 production and urinary nitrogen excretion, with the formula of Brouwer (29). Urine will be collected every 21 h or 27h. Samples will be collected in containers with 10 ml diluted HCl to prevent nitrogen loss through evaporation. The subjects will be told, before entering the respiration chamber, to handle the urine containers with the most caution because of the presence of diluted HCl. The instruction on how to collect urine and handle the containers is attached to the protocol (**attachment 1**). Volume and nitrogen concentration will be measured, the latter using a nitrogen analyzer.

<u>Sleep quality</u>

To determine sleep duration and sleep quality continuously EEG measurements will take place. Sleep quality represents the amount of minutes in which the subject is

either in sleep phase III, IV plus Rapid eye movement sleep (REM) divided by total duration of sleep. Sleep will be registered using 6 electrodes on the head to measure brain activity (EEG), 2 electrodes on the chin to measure muscle activity (EMG), 2 electrodes around the eyes to measure eye movement (EOG) and 2 electrodes on the chest to measure heart rate and cardiac function (ECG). The subject is only allowed to sleep on the times stated by the investigator, so daytime naps and sleeping in is not allowed, and will be controlled via the EEG measurements. To avoid problems regarding sleep no subjects will be included with known sleep problems (such as insomnia, daytime hypersomnia, or repeated waking up during the night). If the subject is not able to sleep at the times stated by the investigator, he has to lie in bed, with the lights off, and rest with his eyes closed. The subject is not allowed to perform other activities during the times he should be asleep. When the subject has not slept for at least 3 hours during the times stated by the investigator, the test will be terminated to reduce the strain for the subject.

<u>Mood, feelings of hunger and satiety</u>

Appetite and mood profiles will be measured hourly and before and after each meal, by visual analogue scales (VAS) (19) and by Profile of Mood States (POMS) and State Trait Anxiety Index (STAI) questionnaires (20, 21).

Aspects of appetite will be assessed using 100 mm VAS with questions about feelings of hunger, satiety, thirst, and desire to eat. Opposing extremes of each feeling are described at either end of the 100-mm horizontal line, and subjects mark the line to indicate how they feel at that moment.

The POMS questionnaire contains 70 adjectives that are rated on a five-point scale, anchored by “much like this” to “much unlike this” and is divided into five subscales (depression, tension, confusion, fatigue and anger), each scoring a maximum of 35. An increase in POMS scores is associated with a worsening in mood, except in the case of “vigor”.

The STAI state questionnaire refers to the transitory emotional response involving unpleasant feelings of tension and apprehensive thoughts. The STAI scale is composed of 20 questions rated on a four-point scale, ranging from “much like this,” to “much unlike this” and requires that subjects describe how they feel generally, on the anxiety-trait scale, and how they feel at a specific moment, on the anxiety-state

scale. The questionnaire can score a maximum of 80 and an increase in STAI state scores is associated with an increase in anxiety.
Filling out the questionnaires will take about a minute per questionnaire, in total 3 minutes. Examples of the questionnaires are attached to the protocol.

<u>Food choice and energy intake</u>

Our subjects are in overall energy balance, as they are weight stable for at least 3 months and not currently on a diet. Also during their stay in the chamber, they will be fed in energy balance (17). Subjects will have their breakfast, lunch and dinner at set time points (**figure 1**). The energy content of the food will be calculated using the equation of Harris-Benedict that calculates Basal Metabolic Rate (BMR) (17). For the first day BMR will be multiplied by an activity index of 1.4. To determine the appropriate level of energy intake to attain energy balance in the respiration chamber, the sleeping metabolic rate (SMR) will be calculated during the first night and multiplied by an activity index of 1.4. The selected activity index is based on results of a study in which physical activity was determined in confined conditions (a respiration chamber) resulting in a mean PAL of 1.4 (calculated as 24-h energy expenditure/SMR) (32).

Energy intake will be divided over the meals as 20% for breakfast, 40% for lunch, and 40% for diner. The macronutrient composition of the diet will be 12/55/33 En% (protein/carbohydrate/fat). Menus -including amount of food in grams- for two different men, who have a different age/weight/height are represented in **figure 2AB**. The diet consists of normal food products, available in the Albert Hein supermarket. The food products will be bought by the investigators and immediately after purchase, transported by car to the university. Refrigerated products will be taken in Deepfreeze bags. After arrival at the university the refrigerated foods will be stored immediately at 4 C° fridge and the bread will be deep-frozen at -20 C° freezer of a research kitchen from the Department of Human Biology. Before preparation the expiration dates will be checked and the investigators will go to the store each week to get fresh products. All foods will be prepared in a research kitchen from the Department of Human Biology by one of the researchers. Further information can be found in the guideline (**attachment 2**) 'MEC azM/Um for preparation, delivery and administration of products for human usage in clinical studies'.

Figure 2A

Relative energy intake for a male subject, aged 30y, height 1.80m, weight 77 kg, calculated using the equation of Harris-Benedict that calculates Basal Metabolic Rate. BMR will be multiplied by an activity index of 1.4.

Ontbijt	Gram
volkorenbrood	77.97
pate	13.60
halva jam	22.27
ontbijtkoek volkoren	27.84
halvarine (40% vet)	5.57
appel	167.07
sultana	34.33
Lunch	
volkorenbrood	116.94
halvarine	16.71
gebraden gehakt	33.41
pate	13.59
ontbijtkoek volkoren	31.71
cup a soup champignon/ham	194.96
halfvolle melk	111.40
appel	167.07
sultana	34.33
Diner	
kant-en-klaar boerenkool	556.94
rauwkost	167.10
honing/mosterd dressing	44.55
sinaasappelsap	250.61

.

Figure 2B

Relative energy intake for a male subject, aged 25 y, height 1.77 m, weight 65 kg, calculated using the equation of Harris-Benedict that calculates Basal Metabolic Rate. BMR will be multiplied by an activity index of 1.4.

Ontbijt	gram
volkorenbrood	71.41
pate	12.45
halva jam	20.40
ontbijtkoek volkoren	25.50
halvarine (40% vet)	5.10
appel	153.00
sultana	31.43
Lunch	
volkorenbrood	107.09
halvarine	15.30
gebraden gehakt	30.60
pate	12.44
ontbijtkoek volkoren	29.04
cup a soup champignon/ham	178.54
halfvolle melk	102.02
appel	153.00
sultana	31.43
kant-en-klaar boerenkool	510.03
rauwkost	153.02
honing/mosterd dressing	40.80
sinaasappelsap	229.50

The rewarding value of food parameters: wanting and liking

To reveal whether circadian misalignment alters rewarding value of food parameters, the subjects have to play a wanting and liking test on a computer (22) before and after diner on the last day of the 3 circadian cycles. The computer test was developed to determine rewarding value, i.e. 'liking' and 'wanting', for 72 items divided in six categories (bread, filling, drinks, dessert, sweets, stationery). 'Liking' was measured by indicating relative preference of paired items (within/between categories), 'wanting' by working to earn items to choose from via a memory game in which the subjects show their preference for a certain food type. Completion of the liking and wanting parts will take about 30 minutes.

Endocrinological aspects

To investigate whether circadian misalignment affects the endocrinological parameters per cycle 6 blood samples (8.5 ml per sample) will be taken, via a catheter inserted into an ante-cubital vein of the contra-lateral arm. The samples will be drawn before and after the meals. From the blood samples the following parameters will be determined melatonin, leptin, glucose, insulin, ghrelin, and GLP-1. These parameters will be studied as their secretion is connected to the circadian rhythm, and we hypothesize that circadian misalignment will alter the concentrations of these parameters (10, 33-35). The amount blood necessary per sample per parameter is shown in **table 1**. The amounts stated are the minimal amounts of blood necessary to determine parameter concentrations through RIA analysis. The total amount drawn during the whole experiment will be 306 ml per subject.

Table 1

**Hormone**	Amount per sample (ml)
Melatonin	2
Leptin	0.5
Glucose	0.5
Insulin	0.5
Ghrelin	3
GLP-1	2
**Total (ml)**	8.5

<u>Body temperature</u>

The body temperature may influence quality and quantity of sleep (18). Whether there is a relation between body temperature and circadian misalignment, will be assessed with a CorTemp™ Data Recorder (HQInc., Palmetto (FL), USA). Subjects will swallow a “thermometer pill” (CorTemp™ Ingestible Core Body Temperature Sensor) that wirelessly transmits core body temperature as it travels through the digestive tract. The sensor’s signal passes harmlessly through the body to the CorTemp™ Data Recorder that is worn on the outside of the body.

4. Withdrawal of individual subjects

Subjects can leave the study at any time for any reason if they wish to do so without any consequences. The investigator can decide to withdraw a subject from the study for urgent medical reasons.

5. Replacement of individual subjects after withdrawal

If subjects leave the study, new subjects will replace them.

8. Safety reporting

1. Section 10 WMO event

In accordance to section 10, subsection 1, of the WMO, the investigator will inform the subjects and the reviewing accredited METC if anything occurs, on the basis of which it appears that the disadvantages of participation may be significantly greater than was foreseen in the research proposal. The study will be suspended pending further review by the accredited METC, except insofar as suspension would jeopardise the subjects' health. The investigator will take care that all subjects are kept informed.

2. Adverse and serious adverse events

Adverse events are defined as any undesirable experience occurring to a subject during the study, whether or not considered related to the experimental treatment. All adverse events reported spontaneously by the subject or observed by the investigator or his staff will be recorded. A serious adverse event is any untoward medical occurrence or effect that at any dose:

- results in death;
- is life threatening (at the time of the event);
- requires hospitalisation or prolongation of existing inpatients' hospitalisation;
- results in persistent or significant disability or incapacity;
- is a congenital anomaly or birth defect;
- is a new event of the trial likely to affect the safety of the subjects, such as an unexpected outcome of an adverse reaction, lack of efficacy of an IMP used for the treatment of a life threatening disease, major safety finding from a newly completed animal study, etc.

All SAEs will be reported through the accredited METC that approved the protocol, within 15 days after the sponsor has first knowledge of the serious adverse reactions.

SAEs that result in death or are life threatening should be reported expedited. The expedited reporting will occur not later than 7 days after the responsible investigator has first knowledge of the adverse reaction. This is for a preliminary

report with another 8 days for completion of the report.

3. Follow-up of adverse events

All adverse events will be followed until they have abated, or until a stable situation has been reached. Depending on the event, follow up may require additional tests or medical procedures as indicated, and/or referral to the general physician or a medical specialist.

9. Statistical analysis

Differences in energy intake and energy expenditure, as well as sleep quality, mood, feelings of hunger and satiety, food choice, and the rewarding value of food between treatments (21 h cycles and 27 h cycle), will be determined using unpaired t-tests. Differences over time and between treatments (21 h cycles and 27 h cycle) will be determined using two-factor ANOVA with repeated measures.

All statistical tests will be two-sided. Differences will be considered significant at $p < 0.05$, and values were expressed as mean ± standard deviation.

10. Ethical considerations

1. Regulation statement

The study will be conducted according to the principles of the Declaration of Helsinki (9th version, October 2008, Seoul). Within this research the Dutch law for medical-scientific research with humans (WMO) is of application.

2. Recruitment and consent

The subjects will be recruited via advertisements on the notice boards of Maastricht University. The subjects will receive a written subject-information and will be informed orally on the study by the investigator. A written informed consent will be obtained from all participants by the investigator. Subjects are allowed to think for one week after they have received the information, before they give approval for participation. They will be told that they can always stop without giving any reason. An independent medical doctor will be present for them to consult.

4. Benefits and risk assessment

This study does not have any benefits for the subjects themselves, but will give possible new knowledge for treatment of obesity.

This study does not include any major risks for the subjects. The relatively extended stay in the respiration chamber (at least 63 h) might put a strain on the subjects, however the subjects have access to a computer, television, radio, dvd-player, telephone, intercom, sink and toilet, and are able to freely move around since there is no movement protocol. Windows are positioned in the door for contact with the researchers, in the wall for an outside view, and between the chambers for visual contact between the subjects. Curtains ensure privacy when needed. Safety precautions include a fire alarm and extinguisher, emergency power and lighting, subject registration on building fire-alert panel and short access to safe escape route. Subjects are also allowed to stop during the study and are able to open the doors to leave the respiration chamber from the inside, if necessary. During the time-blinded period two researchers will always be present during the subjects stay in the respiration chamber, to ensure the safety and wellbeing of the subjects There are no risks for the subjects in using any of the meals because people with certain food

allergies are excluded and all products are regular food items available at the local supermarket (AH). The “thermometer pill” that they have to swallow is completely harmless and runs through the digestive system, where it eventually will exit the body via the feces. Blood sampling in this study is limited and without side effects, apart from its usual risks of minor bruising. Urine sampling is done in urine bottles with 10 ml of diluted HCl, which might pose a risk for the subjects, however they will be carefully instructed on how to sample the urine and handle the urine bottles. The subject needs to follow our schedule on sleeping, filling in questionnaires and eating, however, the majority of the time the subject is awake and he can fill in his own time. The experiment will cost about 144 hours of the subject’s time. Usually, this type of respiration chamber experiments are experienced as very comfortable by the subjects. They are looked after by friendly personnel, served all food prepared for them and experience a pleasant and quiet stay, enabling them to study, read, or work on the computer.

5. Compensation for injury

The investigator has a liability insurance, which is in accordance with article 7, subsection 6 of the WMO.

The sponsor (also) has an insurance, which is in accordance with the legal requirements in the Netherlands (Article 7 WMO and the Measure regarding Compulsory Insurance for Clinical Research in Humans of 23th June 2003). This insurance provides cover for damage to research subjects through injury or death caused by the study.

1. € 450.000,-- (i.e. four hundred and fifty thousand Euro) for death or injury for each subject who participates in the Research;
2. € 3.500.000,-- (i.e. three million five hundred thousand Euro) for death or injury for all subjects who participate in the Research;
3. € 5.000.000,-- (i.e. five million Euro) for the total damage incurred by the organisation for all damage disclosed by scientific research for the Sponsor as ‘verrichter’ in the meaning of said Act in each year of insurance coverage.

The insurance applies to the damage that becomes apparent during the study or within 4 years after the end of the study.

6. Incentives

For participation in this study subjects will receive financial compensation, 65 euro per 24 hours, which is 390 euro in total, as well as transportation fees.

11. Administrative aspects and publication

1. Handling and storage of data and documents

The privacy of the subjects is guaranteed, using subject numbers (for example nr 1) in combination with treatment code (A for 21h treatment and B for 27 h treatment) throughout the study. All subject-characteristics will thus be encoded and only the researcher and his supervisor have access to the code. Subjects can receive their personal results at the end of the study. The collected data will be saved for 15 years; this is the legal period for storage of human documents. All human materials will be removed after publication of the results.

2. Amendments

Amendments are changes made to the research after a favourable opinion by the accredited METC has been given. All amendments will be notified to the METC that gave a favourable opinion.

3. Annual progress report

The investigator will submit a summary of the progress of the trial to the accredited METC once a year. Information will be provided on the date of inclusion of the first subject, numbers of subjects included and numbers of subjects that have completed the trial, serious adverse events/ serious adverse reactions, other problems, and amendments.

4. End of study report

The investigator will notify the accredited METC of the end of the study within a period of 8 weeks .The end of the study is defined as the last patient's last visit.
In case the study is ended prematurely, the investigator will notify the accredited METC, including the reasons for the premature termination.
Within one year after the end of the study, the investigator will submit a final study report with the results of the study, including any publications/abstracts of the study, to the accredited METC.

5. Public disclosure and publication policy

The results will be published in the international peer reviewed literature. The CCMO Statement will be followed, which means that the results will be published within 3 months after analysis.

12. References

1. Haslam DW, James WP. Obesity. Lancet 2005;366:1197-209.
2. Collins CE, Warren J, Neve M, McCoy P, Stokes BJ. Measuring effectiveness of dietetic interventions in child obesity: a systematic review of randomized trials. Arch Pediatr Adolesc Med 2006;160:906-22.
3. Avenell A, Broom J, Brown TJ, et al. Systematic review of the long-term effects and economic consequences of treatments for obesity and implications for health improvement. Health Technol Assess 2004;8:iii-iv, 1-182.
4. Bechtold DA. Energy-responsive timekeeping. J Genet 2008;87:447-58.
5. Swaab DF, Van Someren EJ, Zhou JN, Hofman MA. Biological rhythms in the human life cycle and their relationship to functional changes in the suprachiasmatic nucleus. Prog Brain Res 1996;111:349-68.
6. Spiegel K, Leproult R, L'Hermite-Baleriaux M, Copinschi G, Penev PD, Van Cauter E. Leptin levels are dependent on sleep duration: relationships with sympathovagal balance, carbohydrate regulation, cortisol, and thyrotropin. J Clin Endocrinol Metab 2004;89:5762-71.
7. Spiegel K, Leproult R, Van Cauter E. Impact of sleep debt on metabolic and endocrine function. Lancet 1999;354:1435-9.
8. Mendoza J. Circadian clocks: setting time by food. J Neuroendocrinol 2007;19:127-37.
9. Eng S, Wagstaff DA, Kranz S. Eating late in the evening is associated with childhood obesity in some age groups but not in all children: the relationship between time of consumption and body weight status in U.S. children. Int J Behav Nutr Phys Act 2009;6:27.
10. Scheer FA, Hilton MF, Mantzoros CS, Shea SA. Adverse metabolic and cardiovascular consequences of circadian misalignment. Proc Natl Acad Sci U S A 2009;106:4453-8.
11. Ekmekcioglu C, Touitou Y. Chronobiological aspects of food intake and metabolism and their relevance on energy balance and weight regulation. Obes Rev.
12. Haugen HA, Melanson EL, Tran ZV, Kearney JT, Hill JO. Variability of measured resting metabolic rate. Am J Clin Nutr 2003;78:1141-5.
13. Arble DM, Bass J, Laposky AD, Vitaterna MH, Turek FW. Circadian timing of food intake contributes to weight gain. Obesity (Silver Spring) 2009;17:2100-2.
14. Schoffelen PF, Westerterp KR, Saris WH, Ten Hoor F. A dual-respiration chamber system with automated calibration. J Appl Physiol 1997;83:2064-72.
15. Wijers SL, Saris WH, van Marken Lichtenbelt WD. Cold-Induced Adaptive Thermogenesis in Lean and Obese. Obesity (Silver Spring).

16. Wijers SL, Saris WH, van Marken Lichtenbelt WD. Individual thermogenic responses to mild cold and overfeeding are closely related. J Clin Endocrinol Metab 2007;92:4299-305.
17. Harris JA, Benedict FG. A Biometric Study of Human Basal Metabolism. Proc Natl Acad Sci U S A 1918;4:370-3.
18. Parmeggiani PL. Interaction between sleep and thermoregulation: an aspect of the control of behavioral states. Sleep 1987;10:426-35.
19. Kovacs EM, Lejeune MP, Westerterp-Plantenga MS. The effects of enterostatin intake on food intake and energy expenditure. Br J Nutr 2003;90:207-14.
20. Albrecht RR, Ewing SJ. Standardizing the administration of the Profile of Mood States (POMS): development of alternative word lists. J Pers Assess 1989;53:31-9.
21. Tenenbaum G, Furst D, Weingarten G. A statistical reevaluation of the STAI anxiety questionnaire. J Clin Psychol 1985;41:239-44.
22. Lemmens SG, Schoffelen PF, Wouters L, et al. Eating what you like induces a stronger decrease of 'wanting' to eat. Physiol Behav 2009;98:318-25.
23. Wittmann M, Dinich J, Merrow M, Roenneberg T. Social jetlag: misalignment of biological and social time. Chronobiol Int 2006;23:497-509.
24. Baecke JA, Burema J, Frijters JE. A short questionnaire for the measurement of habitual physical activity in epidemiological studies. Am J Clin Nutr 1982;36:936-42.
25. Stunkard AJ, Messick S. The three-factor eating questionnaire to measure dietary restraint, disinhibition and hunger. J Psychosom Res 1985;29:71-83.
26. Brondel L, Romer MA, Nougues PM, Touyarou P, Davenne D. Acute partial sleep deprivation increases food intake in healthy men. Am J Clin Nutr.
27. Westerterp-Plantenga MS, Lejeune MP, Smeets AJ, Luscombe-Marsh ND. Sex differences in energy homeostatis following a diet relatively high in protein exchanged with carbohydrate, assessed in a respiration chamber in humans. Physiol Behav 2009;97:414-9.
28. Veldhorst MA, Westerterp-Plantenga MS, Westerterp KR. Gluconeogenesis and energy expenditure after a high-protein, carbohydrate-free diet. Am J Clin Nutr 2009;90:519-26.
29. Brouwer E. On simple formulae for calculating the heat expenditure and the quantities of carbohydrate and fat oxidized in metabolism of men and animals, from gaseous exchange (Oxygen intake and carbonic acid output) and urine-N. Acta Physiol Pharmacol Neerl 1957;6:795-802.
30. Levine J, Melanson EL, Westerterp KR, Hill JO. Tracmor system for measuring walking energy expenditure. Eur J Clin Nutr 2003;57:1176-80.
31. Westerterp KR, Wilson SA, Rolland V. Diet induced thermogenesis measured over 24h in a respiration chamber: effect of diet composition. Int J Obes Relat Metab Disord 1999;23:287-92.
32. Westerterp KR, Kester AD. Physical activity in confined conditions as an indicator of free-living physical activity. Obes Res 2003;11:865-8.
33. Benloucif S, Guico MJ, Reid KJ, Wolfe LF, L'Hermite-Baleriaux M, Zee PC. Stability of melatonin and temperature as circadian phase markers and their relation to sleep times in humans. J Biol Rhythms 2005;20:178-88.
34. Mingrone G, Nolfe G, Gissey GC, et al. Circadian rhythms of GIP and GLP1 in glucose-tolerant and in type 2 diabetic patients after biliopancreatic diversion. Diabetologia 2009;52:873-81.

35. Natalucci G, Riedl S, Gleiss A, Zidek T, Frisch H. Spontaneous 24-h ghrelin secretion pattern in fasting subjects: maintenance of a meal-related pattern. Eur J Endocrinol 2005;152:845-50.
